# Predicting diarrhoea outbreaks with climate change

**DOI:** 10.1371/journal.pone.0262008

**Published:** 2022-04-19

**Authors:** Tassallah Abdullahi, Geoff Nitschke, Neville Sweijd

**Affiliations:** 1 Department of Computer Science, University of Cape Town, Cape Town, Western Cape, South Africa; 2 Applied Centre for Climate and Earth Systems Science, Council for Scientific and Industrial Research, Cape Town, South Africa; Newcastle University, UNITED KINGDOM

## Abstract

**Background:**

Climate change is expected to exacerbate diarrhoea outbreaks across the developing world, most notably in Sub-Saharan countries such as South Africa. In South Africa, diseases related to diarrhoea outbreak is a leading cause of morbidity and mortality. In this study, we modelled the impacts of climate change on diarrhoea with various *machine learning* (ML) methods to predict daily outbreak of diarrhoea cases in nine South African provinces.

**Methods:**

We applied two *deep Learning* DL techniques, *Convolutional Neural Networks* (CNNs) and *Long-Short term Memory Networks* (LSTMs); and a *Support Vector Machine* (SVM) to predict daily diarrhoea cases over the different South African provinces by incorporating climate information. *Generative Adversarial Networks* (GANs) was used to generate synthetic data which was used to augment the available data-set. Furthermore, *Relevance Estimation and Value Calibration* (REVAC) was used to tune the parameters of the ML methods to optimize the accuracy of their predictions. *Sensitivity analysis* was also performed to investigate the contribution of the different climate factors to the diarrhoea prediction method.

**Results:**

Our results showed that all three ML methods were appropriate for predicting daily diarrhoea cases with respect to the selected climate variables in each South African province. However, the level of accuracy for each method varied across different experiments, with the deep learning methods outperforming the SVM method. Among the deep learning techniques, the CNN method performed best when only real-world data-set was used, while the LSTM method outperformed the other methods when the real-world data-set was augmented with synthetic data. Across the provinces, the accuracy of all three ML methods improved by at least 30 percent when data augmentation was implemented. In addition, REVAC improved the accuracy of the CNN method by about 2.5% in each province. Our parameter sensitivity analysis revealed that the most influential climate variables to be considered when predicting outbreak of diarrhoea in South Africa were *precipitation*, *humidity*, *evaporation* and *temperature* conditions.

**Conclusions:**

Overall, experiments indicated that the prediction capacity of our DL methods (*Convolutional Neural Networks*) was found to be superior (with statistical significance) in terms of prediction accuracy across most provinces. This study’s results have important implications for the development of automated early warning systems for diarrhoea (and related disease) outbreaks across the globe.

## Introduction

Diarrhoea is a major health concern and has remained among the top leading cause of global morbidity and mortality amongst all ages [1, 2]. Annually, over 2.5 million deaths attributed to diarrhoea is recorded worldwide [3]. The World Health Organization reported that the Sub-Saharan Africa (SSA) and South Asia regions account for more than 80 percent of total world records [1, 3]. Over the SSA region, South Africa is one of the most affected countries. In 2010 and 2015, diarrhoea was reported to be among the top ten leading causes of years of life lost among South African residents [4]. Diarrhoea also accounts for three percent of the total death records in individual of all ages in the country [5]. Some studies such as [6, 7] have shown that diarrhoea infections in South Africa are attributed to nosocomial infections or community acquired resulting from contaminated food and water caused by a range of pathogens. However, studies by [8, 9] reported that climate factors and weather variability influence the level of abundance and seasonality of the pathogens present in the environment, thus the prevalence of diarrhoea can be linked to extremities from weather events.

South Africa is a region that experiences significant temperature and precipitation anomaly, which are factors that play a vital role in the long-term trends of diarrhoea [10, 11]. For example, in Western Cape province of South Africa, the rate of diarrhoea hospitalizations was strongly linked to increase in minimum and maximum temperature [7]. A study in Limpopo province showed that seasons when precipitation rate was below normal coincides with a high number of diarrhoea cases [9]. Thus, the development of a model with the ability to capture complex relationships and long-term dependencies between climate factors and diarrhoea may be effective for diarrhoea predictive analysis. A diarrhoea predictive model could be used for public health surveillance as it will offer timely detection and prompt notification for the control of diarrhoea outbreak.

Several studies have developed models for investigating diarrhoea outbreak in various communities. A vast majority were developed with statistical models such as *Auto-regressive Integrated Moving Average Model* (ARIMA) [12], Poisson Regression [7], *Auto-regressive Analysis of Covariance* Model (ANCOVA) [13] and Time-series Log Linear Regression [8]. For instance, a study by [12] used the influence of climate variables to develop an ARIMA model that predicts the daily incidence of diarrhoea in Beijing. The Poisson Regression model was also used by [7] to assess the relationship between diarrhoea cases and temperature variability in South Africa. Although these studies have proven useful, other studies such as [14, 15] have shown that traditional statistical models and frameworks are often limited for the analysis of high dimensional, imbalanced, and non-linear data. In addition, these studies [14, 15] reported that the limitations of statistical models can be addressed using *Machine Learning* (ML) methods. ML methods are known for their ability to handle high-dimensional data and model complex predictive problems.

Several supervised learning-based ML techniques such as *Support Vector Machines* (SVMs) [16] and Deep learning techniques such as *Convolutional Neural Networks* (CNNs) [17], *Long Short-Term Memory Networks* (LSTMs) [18] have been applied in medical research for developing predictive and diagnostic models for various diseases [14, 15]. For example, CNNs have been used for the detection of Malaria parasite [19] and Tuberculosis diseases [20] in individuals. LSTMs have also been used to predict the outbreak of diseases like Typhoid, Chicken Pox and Scarlet Fever [14]. SVMs were also used for Hepatitis disease detection [21]. These ML methods are widely used for modelling infectious diseases because of the numerous advantages they possess. For instance, CNNs are popular for their powerful feature extraction capabilities [17]. LSTMs are commonly used to handle sequential tasks such as time series forecasting because of their ability to capture long term dependencies [14]. SVMs are widely accepted for their ability to solve nonlinear regression estimation problems, their non-parametric nature enables them to represent complex and nonlinear functions easily [16].

Despite advances in a range of health-care applications using such predictive-based ML [14, 15, 21], there is a lack of research and data on the efficacy of such predictive ML methods for diarrhoea outbreak prediction in Sub-Saharan Africa. Additionally, the overall task performance of ML algorithms, applied to many health-care applications and more broadly to any predictive classification task, largely depends on the manual tuning and calibration by algorithm designers and experimenters of methodological parameters over the course of several experimental trials [22, 23]. Such manual tuning is often ineffective and significantly limits the full potential of task performance achieved by the ML method, especially for high-dimensional, partially observable, noisy and complex task domains [22], as are typified by the nature of data-sets in many health-care applications including diarrhoea outbreak prediction. Task performance also largely depends on the amount of available training data [24], which is a significant challenge for most predictive ML in health-care applications due to the sensitive and controlled nature of health-care data-sets [25]. The inaccessibility of data adds to the difficulty of method comparison, accuracy, and the advancement of ML as a whole [24, 26].

The overall aim of this study is to ascertain the suitability of various ML methods given various climate factors and synthetic (generative) training data for accurately predicting diarrhoea outbreaks. Specifically, the study aims to elucidate what type of ML method is most appropriate when coupled with specific training and test data-sets (that is, specific climate variables, data-sparseness, data-noise and synthetic data compliment), in order to optimise prediction efficacy. Thus, we compared task-performance of three ML methods (CNNs, LSTMs and SVMs) to ascertain the most suitable method for predicting future number of daily diarrhoea cases in nine South African provinces. The average predictive accuracy of each method was compared across multiple datasets and experiment replications. Given the sparse and noisy nature of the data-sets used for method training and testing, we necessarily augmented the available data (real-world data) with synthetic data generated using *Generative Adversarial Networks* (GANs). GANs were selected as they have been previously demonstrated as effective for generating different types of realistic data [24, 25]. Also, since there was a lack of previous research to guide parameter tuning and calibration for optimising such ML methods applied to diarrhoea outbreak prediction, we used the *Relevance Estimation and Value Calibration* (REVAC) method [27]. REVAC is an evolutionary algorithm design for meta-heuristic parameter tuning, and as such was applied to optimise methodological parameters of the ML methods used in this study. Previous work has demonstrated the effectiveness of REVAC for parameter tuning and attaining optimal algorithm performance across a range of complex, noisy and high-dimensional search spaces [28, 29].

## Methods

### Study population

This study focused on the nine South African Provinces which are: *Western Cape*, *Eastern Cape*, *Northern Cape*, *North West*, *Free State*, *Limpopo*, *KwaZulu Natal*, *Gauteng*, and *Mpumalanga*. Most provinces in South Africa experience rainfall in the summer with the exception of Western Cape. Western Cape has a Mediterranean climate that receives rainfall during winter with an average annual rainfall of 515mm. Provinces such as KwaZulu Natal, Free State and Mpumalanga experience the highest annual rainfall rate which is between 800–1054*mm* while Eastern Cape, Limpopo, Gauteng, Northern Cape, and North West province receive an annual rainfall that is between 400–600*mm*. In terms of temperature conditions, Limpopo, Northern Cape, Mpumalanga and North West provinces usually record the highest temperature with annual averages between 27.1–30°C while the least annual average temperatures which are between 22.1–23.3°C are usually recorded for Western and Eastern Cape provinces.

### Datasets

The datasets used for all experiments consists of nine features categorized into two data subsets: Diarrhoea and a set of eight climate features.

For each province, daily sales records of *Loperamide*, an anti-diarrhoea compound that has been evaluated in the treatment of patients with chronic non-specific diarrhoea in South Africa and other parts of the world was obtained from *Clicks Group Limited*, South Africa (https://www.clicksgroup.co.za/). The data contains a 10-year period of total number of loperamide purchased between November 2008 and March 2018. This data was used as a proxy for diarrhoea cases in the region. In this study, the number of diarrhoea cases per day for a specific province was computed as the number of loperamide sales per day associated with the province. Six-hourly data on *Maximum temperature*, *Minimum temperature*, *Air temperature*, *Specific humidity*, *Potential evaporation rate*, *Precipitation rate*, *Surface pressure*, and *Wind velocity* climate factors for each South African province between the period of November 2008 and October 2019 were obtained from the National centres for *Atmospheric Research* and *Atmospheric Prediction*. Please see (https://psl.noaa.gov/).

*Generative Adversarial Networks* (GANs) [25] were used to generate 20, 000 synthetic time-series samples with 24 time-steps each for the diarrhoea and eight climate data in each province. Data augmentation was performed to have sufficient data for making predictions, where synthetic data was augmented with the real-world data-sets in two ways: *upward augmentation* and *downward augmentation*. When the data-sets were augmented upwards, the training set included a combination of the real-world and synthetic samples, but the test set included only the synthetic data-sets and when the data-sets were augmented downwards, the training set included mainly the synthetic data-sets and the test set included the real-world data-set. Technical details on GAN implementation can be seen in S1 Appendix.

**Fig 1:**
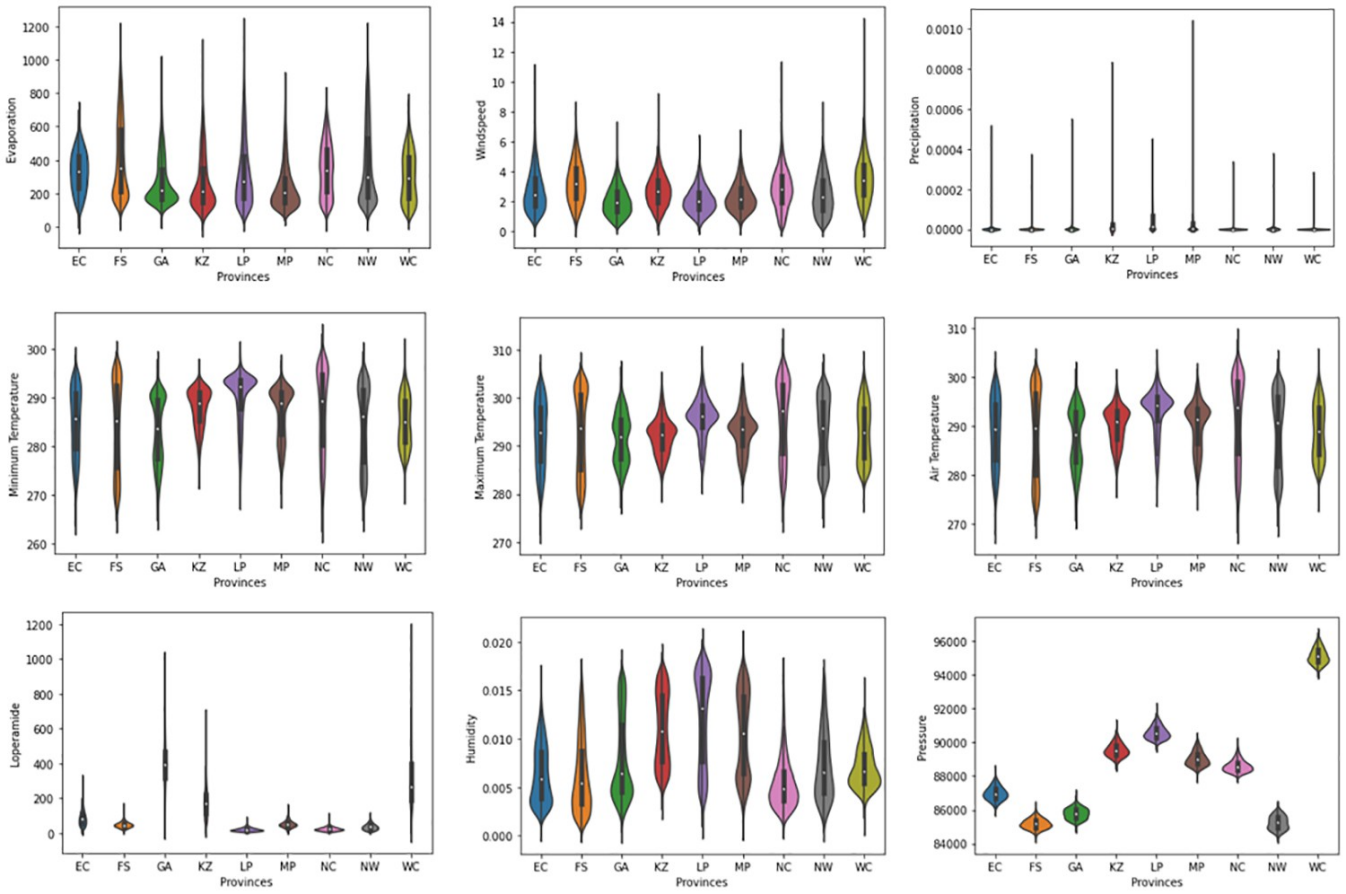
Violin plots showing the distribution of loperamide (diarrhoea) and climate variables across the provinces. EC = Eastern Cape, FS = Free State, GA = Gauteng, KZ = KwaZulu Natal, LP = Limpopo, MP = Mpumalanga, NC = Northern Cape, NW = North West, WC = Western Cape. The distribution of the real-world and synthetic data (augmented data) are shown in S1 and S2 Figs respectively.

The violin plots in Fig 1 show the distribution of the augmented dataset used in the study for each province. The distribution of the diarrhoea case variable (loperamide) is similar across Western Cape, KwaZulu Natal and Gauteng with Western Cape having the highest spread of cases among all provinces. The distribution of the pressure variable is shown to be symmetric across all provinces, meaning that its values occur at regular frequencies while the precipitation variable is positively skewed thus, the mean value for each province is greater than the median. The distribution of the other climate variables is shown to be approximately identical across provinces.

### Data preprocessing

The real world climate and diarrhoea cases data-sets for each province collected for the study were numerical and was ordered in the form of time series. To predict daily diarrhoea cases, the six-hourly climate features data-sets for each province was converted into daily average format. For all experiments, the normalization technique we adopted for our CNNs and LSTMs is the *Min-Max Normalization* because it largely adopted for most neural network regression models [30]. For our SVM methods, we adopted the *Standard Scaling* technique since SVMs assume that the data given as input is within a standard range [31]. We used the python *Scikit-Learn* (https://scikit-learn.org/) library to implement all our normalizations. For all experiments, we divided our data-sets into a ratio of 70: 30 for training and testing our methods. The data-sets with the earlier dates were used for training while the data-sets with later dates were used to test and verify the accuracy of the methods.

### Performance evaluation criteria

To compare and evaluate the performance of our ML methods, the *Root Mean Square Error* (RMSE) was used since it is widely adopted in many prediction studies [14, 15]. The RMSE was also chosen because it is recommended if evaluations based on understanding of predictions are desired [32]. It is also superior at disclosing differences in method task-performance. RMSE is the square root of the mean of the squared differences between actual outcomes and the predictions made by a given method. It is calculated using the equation below:

$$\begin{array}{c} RMSE=\sqrt{\left(\frac{1}{n}\right)\sum_{i=1}^{n}{\left(x_{i}-y_{i}\right)}^{2}} \end{array}$$ (1)

In Eq (1), *x*_*i*_ is the actual value while *y*_*i*_ is the predicted value and n is the total number of observations to be analysed. The ML method with the smallest RMSE error is considered to be the best performing method in terms of prediction accuracy.

### Configuration of ML methods

This study adopted two popular deep learning methods namely CNNs, LSTMs and a traditional ML method SVM for all experiments. These methods were chosen because of their success in time series predictive tasks such as [14, 33]. Asides the powerful feature representation capabilities of deep learning models, the LSTM network is a powerful technique for analyzing temporal data. While the existence of other traditional ML methods such as decision trees [34] and ARIMA [12] are known, SVM was chosen because it is a widely used nonlinear regression estimation technique [16]. In addition, our preliminary analysis showed that the chosen ML methods outperforms the decision trees (see the S1 Appendix section). The rest of this section provides details on how the chosen methods were implemented.

#### CNN method

**Fig 2:**
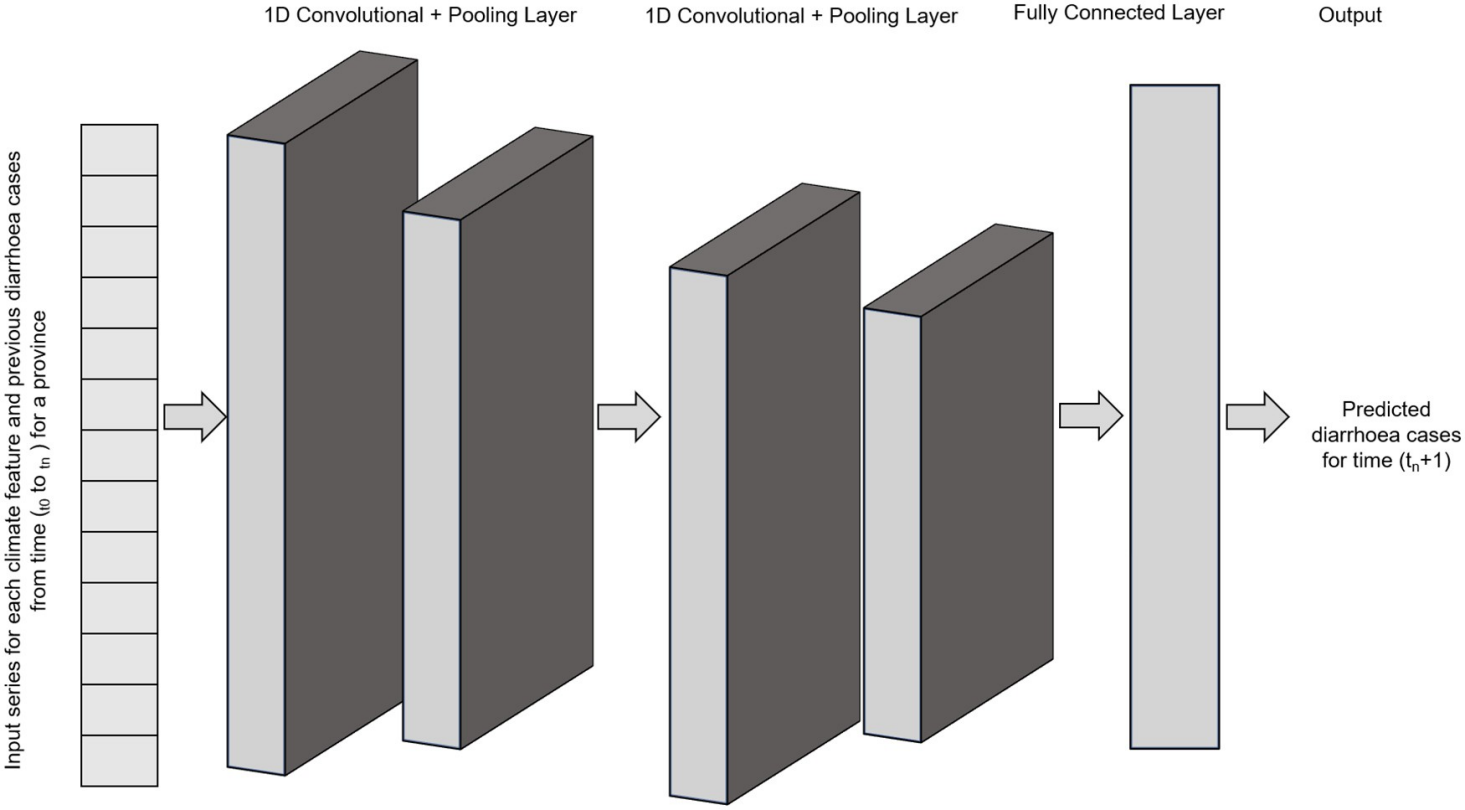
Basic architecture of the *Convolutional Neural Network* (CNN) with two convolution and pooling layers.

*CNNs* are a class of feed forward, deep neural network that consist of multiple convolutional and activation layers, pooling layers, and a fully connected layer as shown in Fig 2. These layers are designed to perform specific tasks in order to extract important features from the input data. After several iterations of convolutions, node activations and pooling the final output is computed in the fully connected layer of the network. Our CNN method was designed with 1D convolutions to match the sequential nature of our input data.

#### LSTM method

**Fig 3:**
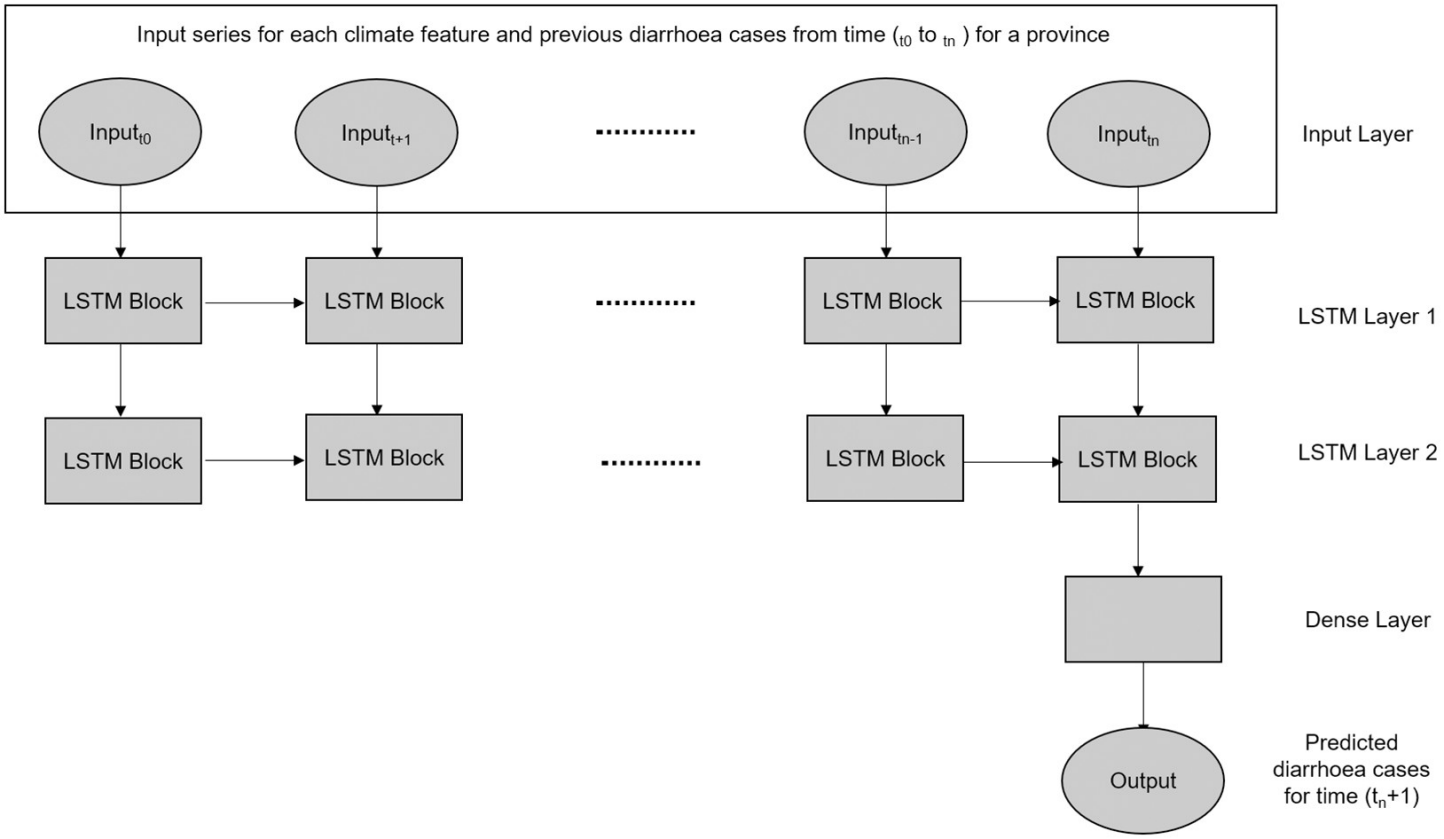
Basic structure of the *Long-term Short Term* (LSTM) method with two LSTM layers.

*LSTMs* as shown in Fig 3 are examples of Neural Networks under the category of *Recurrent Neural Networks* (RNNs) that address the issue of exploding and vanishing gradients. They contain memory cells that maintain their state overtime. The memory cells are managed by gating units that control how it memorize, erase, and expose information. These gating units are called the input gate, forget gate and output gate respectively.

#### SVM method

**Fig 4:**
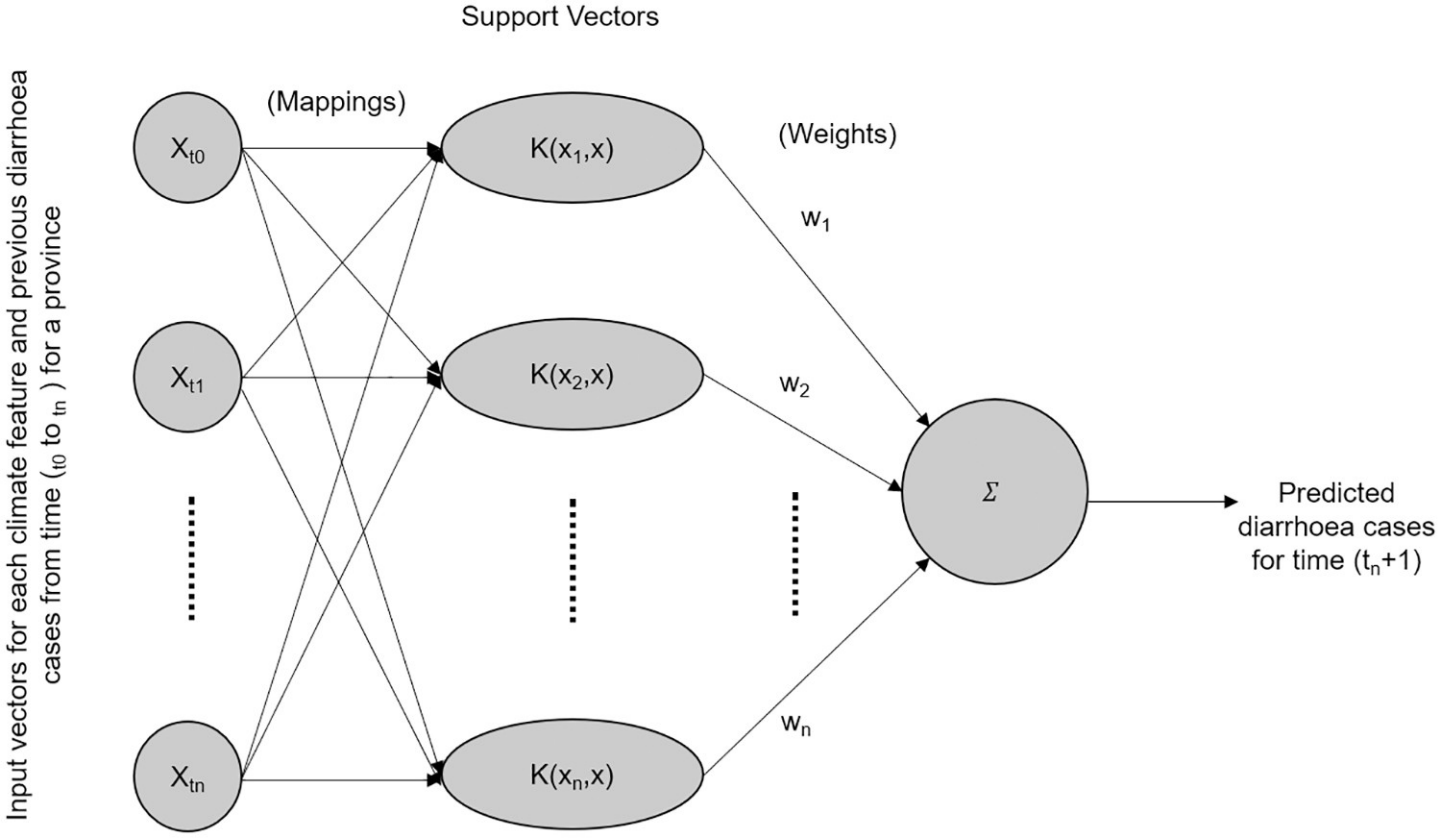
Structure of the *Support Vector Machine* (SVM) regression method. The mappings of the input vectors and the final output is discerned with the RBF kernel function.

*SVMs* are mathematical models whose main function is to find hyper-planes capable of creating margins that separates data points in a high dimensional feature space with the smallest structural risk using kernel functions. We used the *Scikit-Learn package* (https://scikit-learn.org/) to develop all our SVM method with a *Radial Basis Function Kernel* (RBF) for predictions as shown in Fig 4.

For all the methods used in this study, we prepared our input data in a lag format (described in the experiment section), no manual feature extraction step was conducted. Both deep learning methods (CNN and LSTM) were implemented with the *Keras and TensorFlow* (https://keras.io/) deep learning library. The methods were configured to make reproducible results thus, a fixed random seed (https://www.tensorflow.org/) was set for all experiments. For all ML methods, we kept some parameters fixed (based on parameter values established in previous related work [14, 15]), while others were tuned. See Table 1 for the list of tuned parameters.

**Table 1:** Experiment parameters and corresponding value ranges.

ML method	Parameter	Parameter range
SVM	C	[1, 100]
	Gamma	[0.001, 0.1]
LSTM	Dropout rate	[0.1, 0.2, 1.0]
	LSTM layers	[1, 2, 3]
	Neurons	[6, 12, 16, 18, 24, 28, 32, 50, 64, 100]
	Batch size	[4, 16, 18, 32, 64]
	Learning rate	[0.001, 0.01]
	Epochs size	[40, 50, 60, 70, 100, 120, 150, 200]
CNN	Pool size	[1, 2]
	Convolutional layers	[1, 2, 3]
	Kernel size	[1, 2, 3]
	Batch size	[4, 16, 18, 32, 64]
	Learning rate	[0.001, 0.01]
	Epochs size	[40, 50, 60, 70, 100, 120, 150, 200]
	Filter size	[6, 12, 16, 18, 24, 28, 32, 64]

### Determining the optimal ML parameters

One of the major factors that influence the performance of ML methods is the configuration settings of its parameters. Thus, in this study we used grid-search tuning [22] and REVAC [27] parameter tuning methods to find optimal parameter values for all ML methods. Both parameter tuning technique select a combination of possible parameter values from a range of values specified by a user. See Table 1 for the list and range of parameters values specified for each ML method. The deep learning parameters that were not specified used the default values of the *Keras* package. The grid-search method was implemented with the python *Scikit-Learn package* (https://scikit-learn.org/) while REVAC tuning was designed based on the methodology used by Nannen & Eiben [27]. REVAC was implemented at a layer that aids in searching for optimal parameter values for an ML algorithm trying to solve the problem of predicting daily diarrhoea cases. See S1 Appendix for technical details on REVAC implementation. The parameter tuning of each ML method was implemented separately for each province.

### Experiments setup

**Fig 5:**
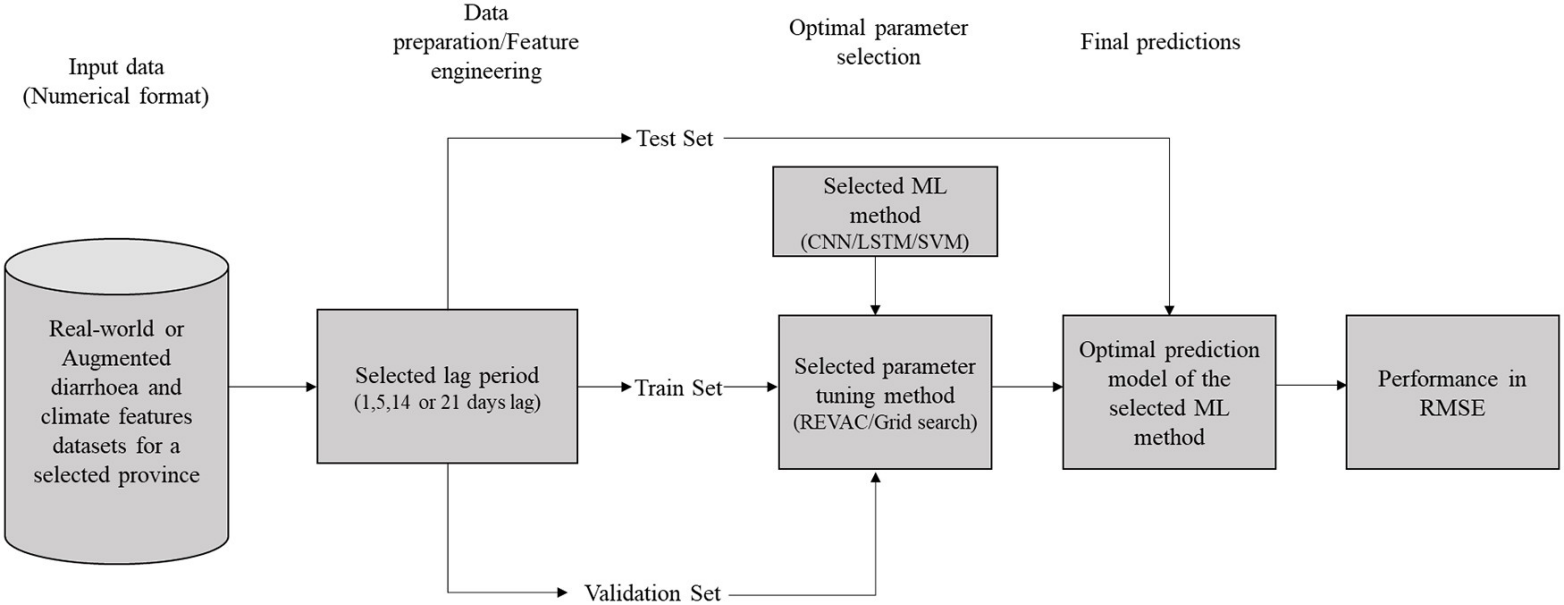
Pipeline of the daily diarrhoea prediction model.

Table 2 gives an overview of the experiments conducted for this study and Fig 5 presents the overall pipeline used to predict daily diarrhoea cases in our experiments. Since this is a regression task, the input data were all in numerical format. Previous studies such as [12, 15] have shown that the basic form of feature engineering applied to a time series prediction task is taking past observations into consideration. Although, deep learning methods are known for automatic feature engineering [17], we applied this feature engineering step across all models for consistency. This approach is also consistent with previous works such as [14, 15, 33]. To make forecasts on the possible number of daily diarrhoea cases, we considered past observations (lags) in all our methods because patterns of the past are likely to be repeated in the future. We tested the predictions of the three ML methods with respect to four different lag periods from all input features. The lag periods we considered include a lag of one (1) day, lag of five (5) days, lag of two (2) weeks and a lag of three (3) weeks. For example, a lag of one day means that the predictions made by a method for the 6th of January 2018 was made with input variables (for all features) of the 5th of January 2018 while a lag of five days means predictions for the 1st of January 2018 was made with input variables (for all features) of the 1st to the 5th of January 2018. These specific lag periods were chosen since our preliminary analyses show that they produce more accurate predictions.

**Table 2:** Experiments overview.

Experiment description	Parameter tuning technique	Datasets used	Research objective
(**I**) Predictions with real-world data	Grid-search	Real-world data	Determine best predicting method given real-world data
(**II**) Predictions with augmented data	Grid-search	Upward and downward augmented data	Determine the effect of augmented data on predicting performance
(**III**) Predictions with augmented data and REVAC parameters	REVAC tuning	Upward and downward augmented data	Determine the impact of REVAC tuning on predicting performance

Thereafter, optimal parameters were selected and we determined the best performing ML method by comparing the RMSE from the predictions made by the three ML algorithms (with respect to the four lag periods) in three different experiments in which for each ML method, predictions were repeated three times for each lag, across each province and the average RMSE result was computed.

The first experiment (*Experiment I*) was implemented with the real-world case data which contained the diarrhoea cases and eight climate features. The objective was to determine which ML method performs best given the amount of data instances contained in the real-world data-set. In order to obtain optimal training parameter values for each ML method across each province, the grid-search method was used in this experiment. For most deep neural networks, such as the CNNs, the computational complexity, can be computed as O(n2) for both training and inference time, where n is the input dataset size [35]. However for networks that deal with sequential learning such as the LSTM, their learning complexity per time step is O(W), where W is the number of parameters in a standard network [36]. The computational complexity of SVM on the other hand is On3 [37]. This shows that computational complexity of each model is different hence training and test time will also differ. In other to address this, the average run-time of each model was computed for this experiment. (see the S1 Appendix section more details).

After concluding the first experiment, we measured the degree of importance of each climate variable to the best performing diarrhoea prediction method in a specific province by conducting a sensitivity analysis [38]. We adopted the *Backward stepwise* method [38] in which we measured the effect of one variable at a time while keeping the other variables fixed. Sensitivity is then measured by observing changes in the RMSE error of the given method based on the omission of a certain variable. The larger the increase in RMSE, the higher the importance of the omitted variable. The second experiment (*Experiment II*) was conducted to determine the effect of augmented training and testing data as well as the effect of a larger training data size on the prediction performance of the three ML methods. The data-sets used in this experiment were combinations of the synthetic and real-world data-set, that is, the upward and downward augmented data in each province. Predictions by each ML method were made with each input data-set separately for each province. The data preprocessing steps and the parameters selected by the grid-search tuning in the first experiment were maintained for each ML method with regards to a specific province.

The third experiment (*Experiment III*) was performed to determine the effect of REVAC parameter tuning on the prediction performance of the three ML methods with the upward and downward augmented data. The major difference between the second and third experiment is the method used for tuning the parameters of each ML method. For all the prediction tasks carried out in the third experiment, data preprocessing steps taken for the three ML methods were the same as the previous experiments. However, the parameter values of each ML method were tuned with REVAC tuning method. Once the REVAC parameter tuning tasks were completed for each ML method, the fittest set of parameter values for each province were used to carry out final predictions.

## Results

Table 3 represents the average RMSE for predictions made with real-world data in all provinces. We observed that the high performance of the CNN method was closely followed by the LSTM method. SVM on the other hand showed the poorest performance. Table 3 also showed that the CNN method had the least overall RMSE average of 31.55% while LSTM and SVM averages were 32.91% and 33.89% respectively. We can infer from these results that the RMSE errors are lower for the deep learning methods (CNN & LSTM).

**Table 3:** *Root Mean Square Error* (RMSE) averages for predictions using real-world data.

ML method	RMSE
Convolutional Neural Network (CNN)	31.55%
Long-term Short Term Memory (LSTM)	32.91%
Support Vector Machine (SVM)	33.89%
Standard Deviation	0.008

**Fig 6:**
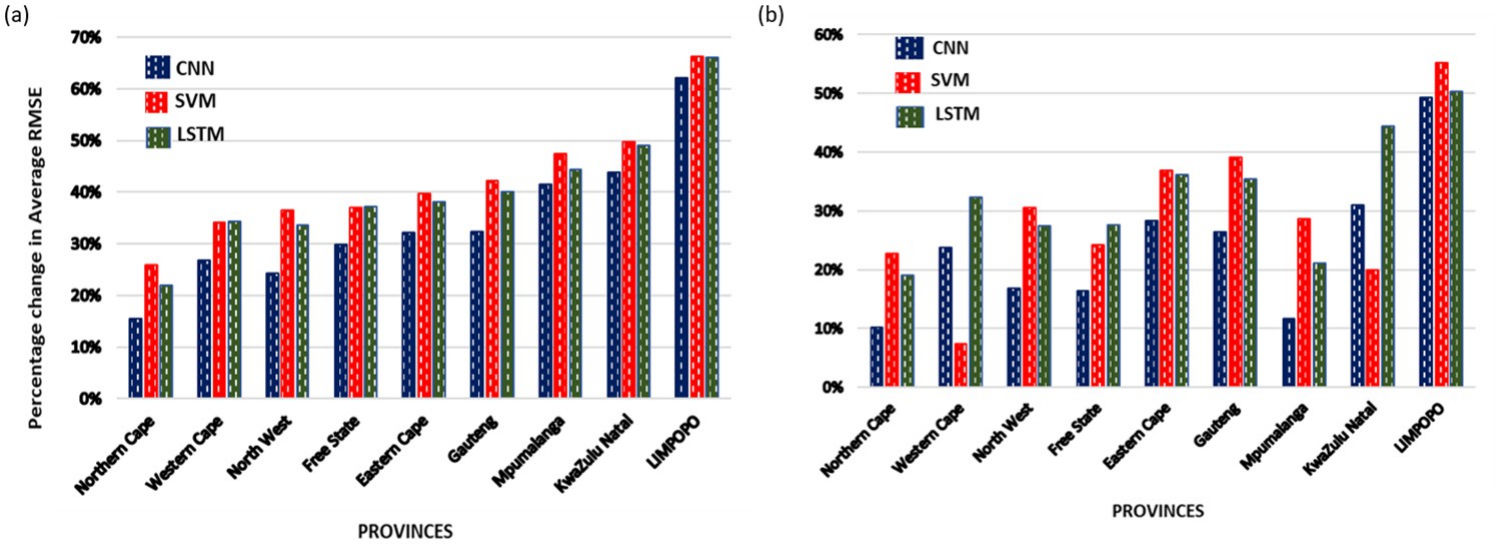
Percentage change in performance of each ML method for predictions in Experiment II. (a) & (b): Percentage change in performance of each ML method over each province when predictions were made with the (a) *upward* augmented data-set and (b) *downward* augmented data-set instead of the *real-world* data. High percentage RMSE indicates an improvement in performance and vice-versa.

Fig 6 shows that the use of augmented data greatly improved the performance of the three ML methods in each province. Predictions for Limpopo province show the highest improvement with over 50% increase for each ML methods when both upward and downward augmented data were used for predictions. However, over most provinces, the percentage increase in performance for predictions with the LSTM and SVM methods was more than the CNN method.

Table 4 compares the performance of the overall predictions made by each ML method when the augmented data-sets were used based on the parameter tuning technique selected. By comparing the RMSE of the augmented data made with grid-search parameters in Table 3 against the RMSE of predictions made with the real-world data in Table 2. For each ML method, the predictions made with the augmented datasets yielded better and lower RMSE than their predictions with the real-world data-sets. Thus, we can infer that the amount of training data used for training, significantly affects the prediction performance of all the three ML methods. By comparing the average RMSE percentages across data-sets, it also shows that CNN outperformed the other methods when the real-world dataset was used alone while LSTM outperformed the other methods when either of the augmented datasets were used.

**Table 4:** RMSE averages for REVAC and grid-search method parameter tuning.

ML Method	REVAC tuning		Grid-search tuning	
	Upward augmented data	Downward augmented data	Upward augmented data	Downward augmented data
CNN	22.07%	23.86%	23.11%	25.80%
LSTM	21.60%	23.61%	21.93%	23.78%
SVM	22.17%	27.30%	22.17%	27.97%
Standard Deviation	0.003	0.021	0.006	0.134

**Fig 7:**
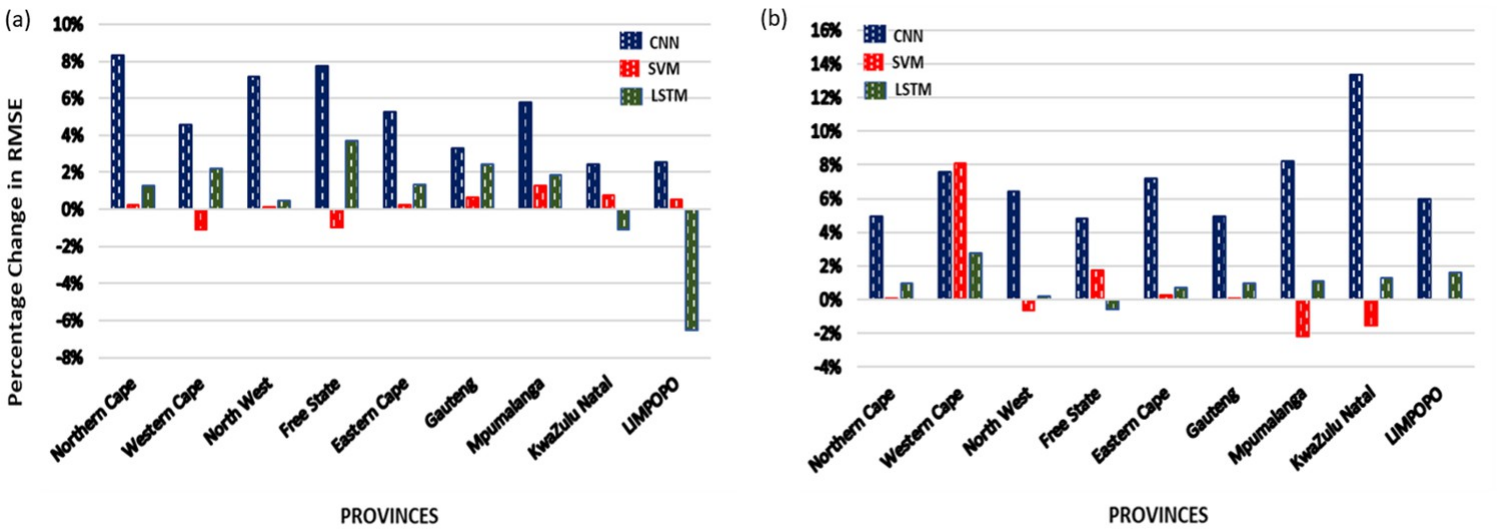
Percentage change in performance of each ML method for predictions in Experiment III. (a) & (b): Percentage change in performance of each ML method over each province when predictions were made with the parameters from REVAC tuning instead of the *grid-search* parameters for (a) *upward* augmented data and (b) *downward* augmented data-set. High percentage RMSE indicates an improvement in task performance and vice-versa.

Fig 7 shows the results when the parameters of the three ML methods were tuned with REVAC instead of grid-search. We found that the CNN method’s prediction results improved across all provinces. The highest percentage increase recorded for CNN was over 12% and the least increase was about 2.5%. The LSTM method’s performance also increased across most province, however, its predictive task performance declined in Limpopo, KwaZulu Natal and Free state provinces. Among the three methods, the SVM recorded the highest number of provinces that saw a decline in task performance. The average increase of SVM task performance across all provinces was also the least.

**Fig 8:**
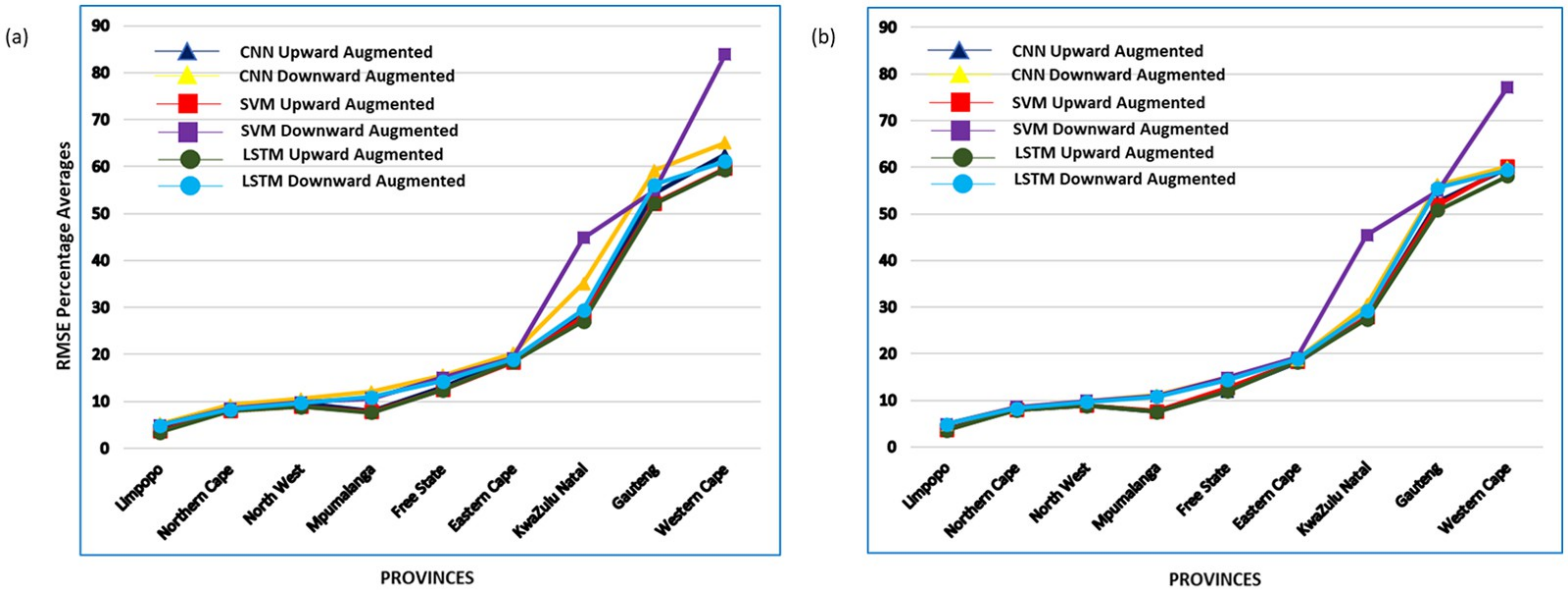
Provincial results of the ML methods with the augmented data-sets in Experiments II & III. (a) & (b): Results of the predictions with the augmented data-sets for each province (a) represents the results with *grid-search* tuned parameters and (b) represents the results with *REVAC tuning* parameters. Low RMSE averages indicate better task performance and vice-versa.

Fig 8 shows the provincial prediction results of the ML methods when augmented data was used for training. In Fig 6a, when grid-search parameters were used, the LSTM method outperformed all the other methods in most provinces with both augmented data-sets and was closely followed by the SVM except in Western Cape and KwaZulu Natal province where the CNN outperformed the SVM. When REVAC tuning parameters were used as shown in Fig 6b, the LSTM method still outperformed the other methods for most provinces and was closely followed by the CNN for most of the data-sets. However, in Gauteng province, the SVM outperformed the CNN.

**Fig 9:**
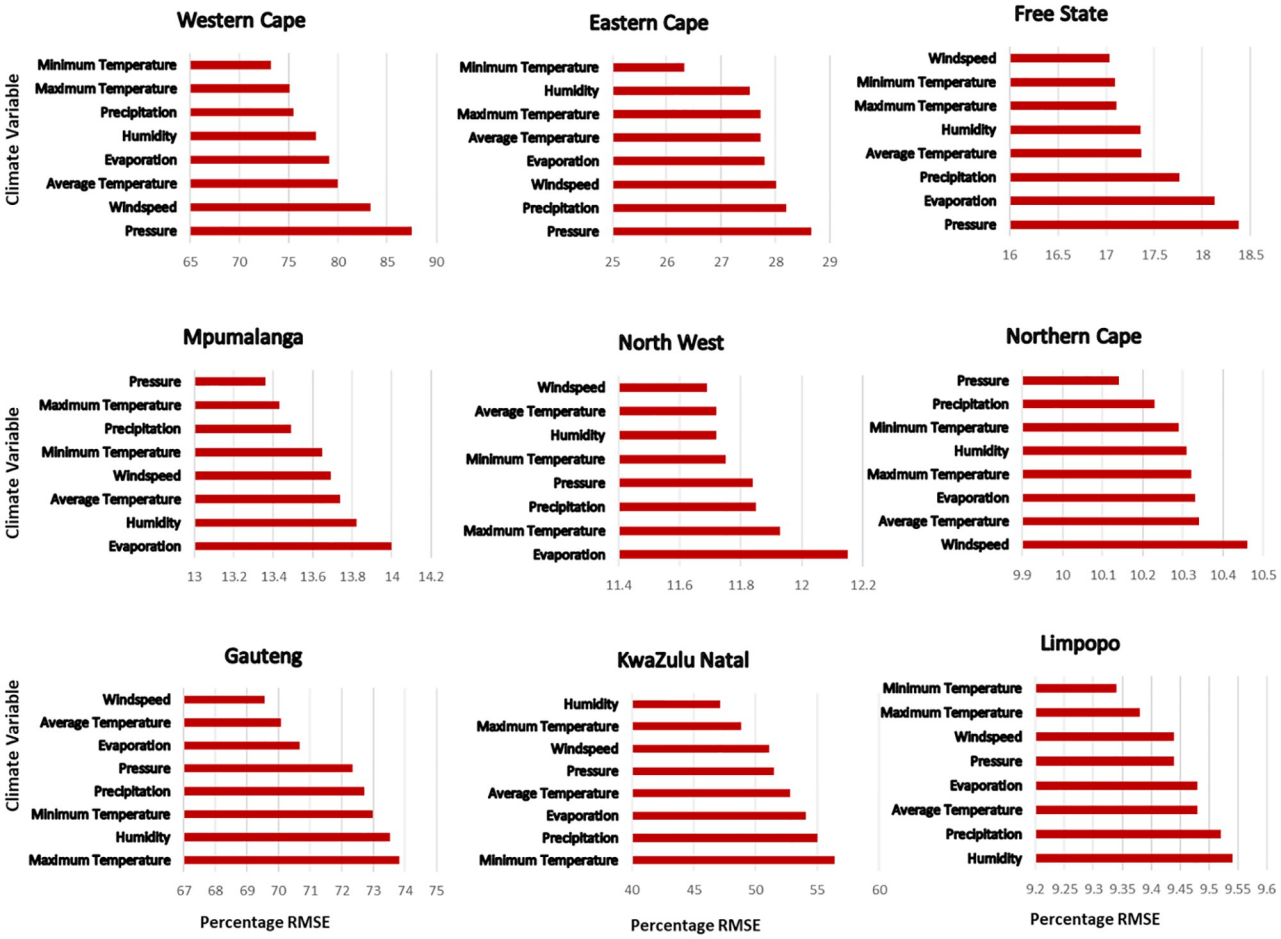
Variable importance plot. Result of the sensitivity analysis carried out for the CNN prediction method for each province. The x-axis indicates the prediction accuracy of the method once the variable on the y-axis is omitted from the method. The longer the bar, the larger the loss in accuracy and the higher the importance of that variable.

The results from the sensitivity study we conducted in Fig 9 shows that the relative importance of each climate variable differs across provinces. For instance, over provinces such as Western Cape, Eastern Cape and Free State, the *Pressure* climate variable was the most sensitive when training any given diarrhoea outbreak prediction method. Whereas, in North West and Mpumalanga, *Evaporation* was the most sensitive climate variable. In Gauteng, Maximum Temperature was most important while in and KwaZulu Natal, *Minimum Temperature* was more sensitive. In Limpopo, *Humidity* was most sensitive variable while *Wind speed* was more important in the Northern Cape.

## Discussion

The results of our experiments revealed that although the *Deep Learning* (DL) methods (Configuration of ML methods section) outperformed the SVM (SVM method section). In most tasks, there was no clear best ML method overall. The ML methods showed different levels of skill based on the availability of training data and the type of parameter tuning method used during training.

### Performance based on dataset type

The CNN method (section CNN method) was able to generalize well and select important features to yield the most satisfactory performance when only real-world data was used for making predictions regardless of its limited training set size. Based on different metrics, some studies [19, 20] have shown results for CNNs to be more accurate than several other methods for infectious diseases prediction. We theorize this to be a result of CNNs being effective universal approximators capable of automatic feature engineering [17]. Our findings also agree with previous research which showed that deep neural networks outperform traditional ML algorithms for most disease prediction tasks [19, 20].

The prediction performance of all ML methods improved when the augmented data-sets were used for training, with the LSTM (LSTM method section) giving the overall best performance. This implies that a large training set size boosts the performance of most ML algorithms. We also surmise that the LSTM method performs better when the size of training data is large, perhaps the reason for its relatively poor performance in the first experiment where only real-world data with limited training set was used. A study conducted by [39] have shown that LSTM benefits from a large training set size. In addition, Another study by [14] reported that LSTMs are a state of the art for capturing the long-term dependencies specific to a given data-set thus their ability to learn patterns in sequential data with sufficient training size regardless of its noisy nature.

### Performance based on parameter tuning method

With respect to the parameter tuning as a factor for task performance with the augmented data, we found that with the given grid-search parameters (Table 1), the average percentage increase in task performance of the CNN method was the least when compared to the other methods across individual provinces. The provincial instances such as in *Gauteng*, *Eastern Cape*, and *Mpumalanga* in Fig 8a (Provincial results of the ML methods with the augmented data-sets in Experiments II & III. figure) where SVM outperformed the CNN method is likely due to the parameter settings of CNN used during training. Therefore, we deduce that the choice of parameters greatly affects the performance of deep learning models especially when applied to noisy and augmented data-sets. Thus, we setup a different experiment with REVAC tuning strategy.

With the REVAC parameter tuning implementation, the CNN method gave the highest percentage increase in performance across each province. However, the LSTM method’s prediction performance was still better than the other methods for most provinces. However, the SVM demonstrated the least average percentage increase and the highest average percentage decrease across the provinces. Therefore, we can infer from these results that the REVAC parameter tuning is not ideal for the SVM method rather it is more suited to deep learning methods. A possible explanation maybe the low dimensional search space of parameters for the SVM method considering that an SVM’s (with RBF kernel) major parameters are gamma and C only. A study by [40] have found that predefining a search space especially for few parameters can be difficult. However, [22] reported that grid-search is better suited for low dimensional search space perhaps the reason for SVM’s satisfactory performance with grid-search tuning.

In Table 5, we compared the performance of the results obtained when REVAC parameter tuning was used on the upward augmented data with the results of some existing models on diarrhoea outbreak prediction with different datasests [14, 15, 41]. Although our RMSE values appear lower, we note that the difference in the error values may be due to the type/size of the dataset used in the different study as well the unit and scale of the dataset.

**Table 5:** Root Mean Square Error (RMSE) performance comparison with the existing diarrhoea prediction studies.

Study	CNN	LSTM	SVM	RF	ARIMA
our study	0.22	0.21	0.22	-	-
[14]	-	1.43	-	-	1.38
[15]	-	-	49.91	48.14	-
[41]	-	-	-	0.45	0.31

### Sensitivity analysis

Our parameter sensitivity analysis (Experiments setup section) demonstrated that the prediction of diarrhoea outbreak by the given ML methods is influenced by specific climate factors. The most prominent (influential) factors are *precipitation*, *humidity*, *evaporation* and *temperature*, although their levels of influence differ across South African provinces. Our findings are in agreement with studies such as [7, 8] that have shown that diarrhoea cases increase for every 1°C increase in temperature. In addition, related work by [42] reported that evaporation rate is strongly linked to high temperature. Since increase in diarrhoea cases have been associated with high temperature, perhaps diarrhoea can also be linked to evaporation rate. Other studies [9, 15] have also demonstrated that *precipitation rate* and *humidity* are strongly related to reported increases in diarrhoea-related hospitalizations.

### Study contributions

A key contribution of this research is the first comprehensive study and application of pertinent ML methods to real-world health-care data sourced from various South African medical institutions in order to formalise an effective predictive machine learning methodology for Sub-Saharan Africa (currently, one of the most adversely affected areas, globally, by diarrhoea outbreaks [1, 3]). A second key contribution of this research is the use of evolutionary optimisation for automating parameter tuning for a given ML method and associated training data-set, as well as demonstration of data augmentation techniques, such as use of generative models to generate artificial data [24, 25] to complement training data deficiencies.

While our study has demonstrated that ML can be used for diarrhoea outbreak prediction with climate factors. The results can be improved in some ways. For example, taking other human and environmental factors that cause the spread of infectious diseases into consideration may improve the accuracy of future diarrhoea prediction models. Given the different strength of each ML algorithm, developing a hybrid method that combines the advantage and benefits of at least two ML algorithms may result in a methodology that yields consistently high predictive task performance regardless of the conditions set in an experiment.

## Conclusion

The global burden of diarrhoea is a major public health problem that causes both personal and widespread harm. This study ascertained the applicability of various *Machine Learning* (ML) methods in the development of automated early warning system for predicting the outbreak of diarrhoea in South Africa given specific climate variables. We compared the predictive task performance of various ML methods, including Support Vector Machines, *Long-Short Term Memory Neural Networks* (*LSTM*) and *Convolutional Neural Networks* (*CNNs*), for predicting daily diarrhoea cases over nine South African provinces. Prediction comparisons were with respect to a specific set of climate variables and varying proportional combinations of real-world and synthetic (data augmentation) training and testing data. Results indicated that overall (for all real-world data-sets), our CNN yielded the highest accuracy predictions supporting the well established predictive capacity and efficacy of deep-learning systems. However, given synthetic training and testing data-augmentation, our *LSTM* yielded the most accuracy predictions overall. This also study elucidated that the climate variables: *precipitation*, *humidity*, *evaporation*, and *temperature*, yielded the greatest impact on daily diarrhoea cases across South Africa, and were thus the data-set variables integral to the predictive success of our tested methods. Thus, a key contribution of this study is the guidance it provides researchers in selecting a suitable ML method for disease outbreak prediction (diarrhoea case prediction in this study), given real-world and augmented training and testing data-sets containing specific types of climate variables. Current research is applying further predictive machine learning methods in an ongoing effort to develop automated early-warning systems for broad-spectrum disease outbreak prediction across various developing nations with deficient public health systems.

## Supporting information

S1 Appendix (PDF)

S1 Fig Violin plots showing the distribution of the upward augmented data for loperamide (diarrhoea) and climate variables across theprovinces. EC = Eastern Cape, FS = Free State, GA = Gauteng, KZ = KwaZulu Natal, LP = Limpopo, MP = Mpumalanga, NC = Northern Cape, NW = North West, WC = Western Cape.
(TIF)

S2 Fig Violin plots showing the distribution of the downward augmented data for loperamide (diarrhoea) and climate variables across theprovinces. EC = Eastern Cape, FS = Free State, GA = Gauteng, KZ = KwaZulu Natal, LP = Limpopo, MP = Mpumalanga, NC = Northern Cape, NW = North West, WC = Western Cape.
(TIF)
