# Supplementary material for: Predicting diarrhoea outbreaks with climate change
Source: PLoS One. 2022 Apr 19;17(4):e0262008. doi: 10.1371/journal.pone.0262008 (PMC9017952; doi:10.1371/journal.pone.0262008)
Supplement: S1 Appendix — (PDF) [file pone.0262008.s001.pdf]

Appendix section

Performance comparison of CNN, LSTM and SVM with Decision Trees

Our experiments with the real-world data showed that decision trees performed poorly across province when compared to the other methods (Table A1). Decision trees recorded the worst performance in Western cape province with a root mean square error (RMSE) of 161.06%. CNN on the other hand, had the best performance across most provinces and was closely followed by the LSTM. The performance of SVM is similar and comparative with the deep learning models especially in the Northern Cape province where it had a RMSE of 10.97% and the CNN and LSTM recorded 10.24% and 10.26% respectively. The parameter settings of the decision trees were the python Sci-kit learn library default while the parameter settings of the CNN, SVM and LSTM methods can be found in the main body of work.

Table A1: Average Root Mean Square Error (RMSE) for each province using real-world datasets

Provinces	CNN RMSE	SVM RMSE	LSTM RMSE	Decision Trees RMSE
**Western Cape**	85.42%	90.58%	90.47%	161.06%
**KwaZulu Natal**	51.03%	55.93%	53.14%	80.67%
**Limpopo**	10.27%	11.00%	9.95%	15.24%
**Free Town**	18.64%	19.94%	19.80%	29.97%
**Mpumalanga**	13.67%	14.92%	13.82%	21.50%
**Northern Cape**	10.24%	10.97%	10.26%	15.34%
**North West**	12.72%	14.08%	13.34%	18.95%
**Gauteng**	80.36%	90.28%	86.93%	148.97%
**Eastern Cape**	28.25%	30.38%	29.77%	48.85%

Technical details for synthetic data generation

We used GANs to generate synthetic data for this study. Synthetic data was generated to augment the real-world datasets for training and testing.

• **Data pre-processing:** To train the GAN, the real-world daily diarrhoea and 8 climate features datasets with a sequence length of 24 was used per province. The datasets were normalized/scaled with the Min-Max scaler python Scikit-learn package with a feature range of (-1,1). After pre-processing, there was 3736 data instances across all 9 features available for training. After synthetic samples were generated, the datasets were reverted to their original scale.

• **GAN Architecture:** The GAN model we used made use of LSTM network for both the generator and the discriminator. The choice of the LSTM network was due to the fact that studies have shown them to be good for learning sequences and our training data was timeseries in nature. The LSTM network we used for our generator had a depth of 3 with 100 hidden units while our discriminator LSTM network had depth of 1 with 100 hidden units as well. Since GANs generates samples from a specific latent space, (a latent variable is an unobserved variable, and a latent space is a multi-dimensional vector space of these variables. The latent dimension is basically the size of the latent space) we tried different latent dimensions ranging from 5 to 70. We noted that larger latent space dimensions generate more realistic looking samples especially with multivariate datasets. The cross-entropy loss was used to measure the performance of the discriminator and generator.

• **Sample Generation:** To generate samples, the GAN model was trained with different batch sizes (8, 16, 32) across different epochs (200,300,400,500) and latent dimensions. To determine if the synthetic samples were close to the original data, visual comparison between the original and synthetic data was done. In addition, we computed the average difference measure between the original and synthetic data to further determine how close the synthetic data is to the original data. In the early stage of learning, the samples were different but as learning progresses further, the model eventually generates realistic looking samples for the diarrhoea and climate features dataset. After training, the GAN model was used to generate 20,000 synthetic samples. These samples were in the form of samples, timesteps (also known as sequence length) and features where each time step can be used as a lag period during prediction experiments. The GAN model was trained separately for each province and for each province, 20,000 synthetic samples with a sequence length of 24 was generated and used for our subsequent experiments. See the GitHub repository (https://github.com/aminalawal/Predicting-Diarrhoea-Outbreak-with-Climate-Change) for the synthetic datasets and GAN implementation source code.

Data Augmentation

To prepare the dataset for predictions with the three ML models, a combination of the synthetic and real-world dataset was made for each province. The data from both the real-world and synthetic set based on a specific lag period were augmented in different proportions as shown in Table A2. The GAN model in this study does not generate Date as a variable rather it generates samples in the form of a series/sequence. Therefore, the two datasets (real-world and synthetic) were combined in two ways explained below.

- **Upward Augmented**: Here, the real-world data was used as the earlier series while the synthetic data follows. When the datasets are augmented this way, the training set will include a combination of the real-world and synthetic samples, but the test set will include only the synthetic dataset.
- **Downward Augmented**: Here, the synthetic data was used as the earlier series while the real-world data follows. When the datasets are augmented this way, the training set will include mainly the synthetic datasets due to its quantity and the test set will include the real-world dataset.

The upward augmented and downward augmented datasets were used separately by the three ML models for prediction across each province in five different combination proportions

shown in Table A2. For example, if a dataset for a specific province is prepared with the 50/50 distribution Table A2, it means that 50% (10,000 samples) of the synthetic data and 50% (1881 samples) of the real-world data will be augmented in both upward and downward manner and will be used separately by an ML model both for prediction. The final prediction result is computed by averaging the results from the prediction from each combination proportion used as input data (this is done separately for upward and downward augmented data).
In addition, after augmentation, 70 percent of the total datasets was used for training while the remaining 30 percent were used as test set. These datasets were used for prediction per province per lag (that is, lag of 1, lag of 5, lag of 2weeks, lag of 3 weeks) in both Experiment II & III.

Table A2: Distribution and proportions of dataset used for predictions in Experiment II & III over each province.

Synthetic Samples (Total size =20,000)	Real-world Samples (Total size = 3,763)
90% (18000)	10% (376)
80% (16,000)	20% (753)
70% (14,000)	30% (1130)
60% (12,000)	40% (1505)
50% (10,000)	50% (1881)

REVAC Parameter Tuning

REVAC is an evolutionary method formally designed to tune the parameters of Evolutionary algorithms. Given an objective, a population of parameter vectors and n number of iterations, REVAC explores, selects, and evaluates a set of possible parameter values. By adopting some concepts of evolution, such as mutation, recombination, selection and replacement, it improves and updates the distribution of the parameter vectors such that after each iteration, there is a high chance of obtaining optimal performance when a combination of those parameters' values is adopted for training an algorithm. The REVAC algorithm adopted for this study was based on the methodology used by Nannen & Eiben [1]. REVAC was implemented at a layer that aids in searching for optimal parameter values for an ML algorithm trying to solve the problem of predicting daily diarrhoea cases. REVAC itself works with a set of parameters that determines how efficiently it runs. The list of REVAC parameters and their values can be seen in Table A3. The list of parameters for each ML algorithm to be tuned was similar to the parameters we tuned with the grid search tuner in the first experiment. To tune the parameters of each ML algorithm these REVAC parameters must be set first. In addition, a specific data set for training and testing must also be provided. In our study, the objective of the REVAC tuner was to minimize a given fitness function which was the RMSE of the predictions made by the ML algorithm for a specific input data. The best parameters yielded by each ML algorithm for the input data based on the REVAC tuner was stored and used later for final predictions.

Table A3: REVAC Parameters used for all ML Models

Parameter	Parameter Range
Population Size	80
No. of Generations	100
No. of Parents for Crossover	2
No. of children to be created per generation	1
No. of parents to be replaced per generation	1

Hardware Specifications

All experiments were conducted on a single machine using an Intel core i7 UHD 620 processor and an NVIDIA MX 150 GPU with a CUDA capability of 6.1. The memory specifications of the machine are an 8GB DDRAM and a 1 TB Hard Disk.

Computation Time

The complex architecture of deep learning models requires a lot of computational resources for completion of most predictive task. The amount of computational resources is also dependent on the size of the input data. Using the hardware specification described above, the average computational training and test time for our experiment with the real-world data across all provinces is shown in Table A4. The SVM recorded the least time overall while the LSTM model recorded the highest computational time. This may be due to the number of sequential computations in the LSTM layer. Studies such as [2] have shown that LSTM models require incurs memory and time complexity especially for large scale tasks. However, issues of computational speed can be addressed by training large models with powerful GPUs.

Table A4: Average computation time for testing and training each ML method with real-world data across provinces

ML Method	Average Computation Time (s)
SVM	3.86
CNN	11.90
LSTM	1279.48

References

1. Nannen V, Eiben AE. Efficient relevance estimation and value calibration of evolutionary algorithm parameters. In 2007 IEEE congress on evolutionary computation 2007 (pp. 103-110). IEEE.

2. Miao Y, Li J, Wang Y, Zhang SX, Gong Y. Simplifying long short-term memory acoustic models for fast training and decoding. In2016 IEEE International Conference on Acoustics, Speech and Signal Processing (ICASSP) 2016 Mar 20 (pp. 2284-2288). IEEE.
