# Supplementary figures and images for: Predicting diarrhoea outbreaks with climate change
Source: PLoS One. 2022 Apr 19;17(4):e0262008. doi: 10.1371/journal.pone.0262008 (PMC9017952; doi:10.1371/journal.pone.0262008)

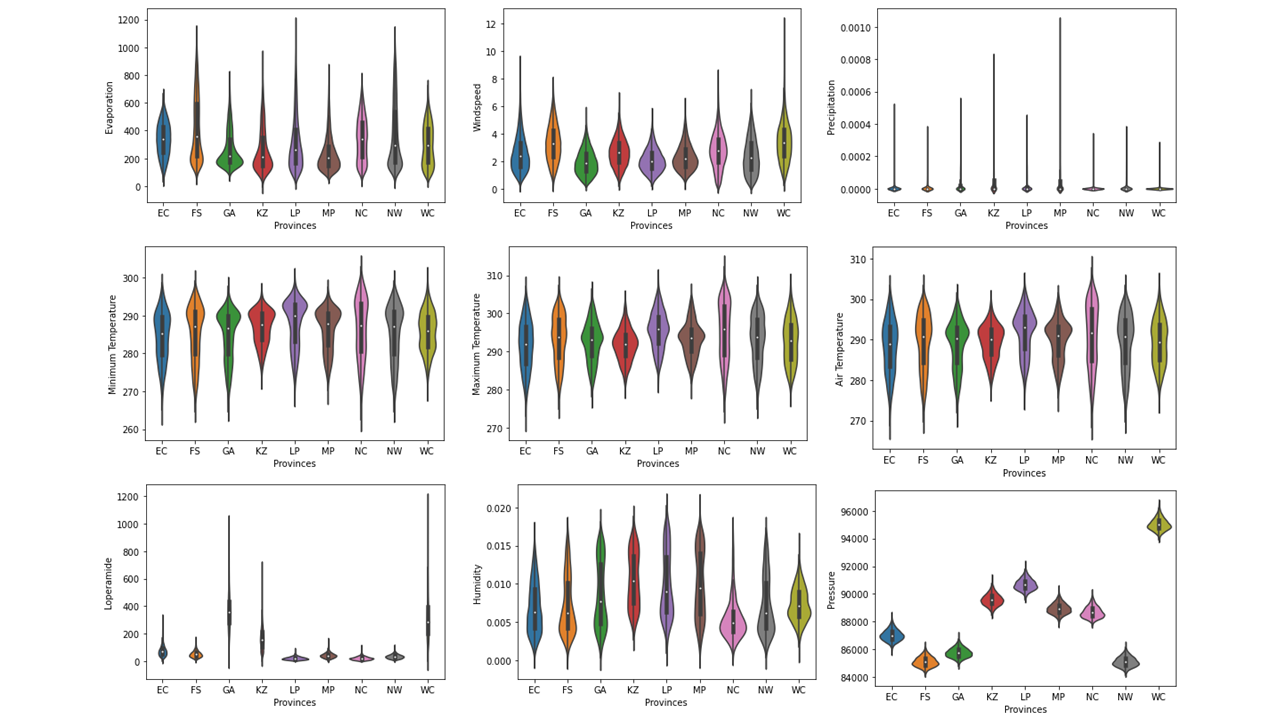

Supplement: S1 Fig — EC = Eastern Cape, FS = Free State, GA = Gauteng, KZ = KwaZulu Natal, LP = Limpopo, MP = Mpumalanga, NC = Northern Cape, NW = North West, WC = Western Cape. (TIF) [file pone.0262008.s002.tif]

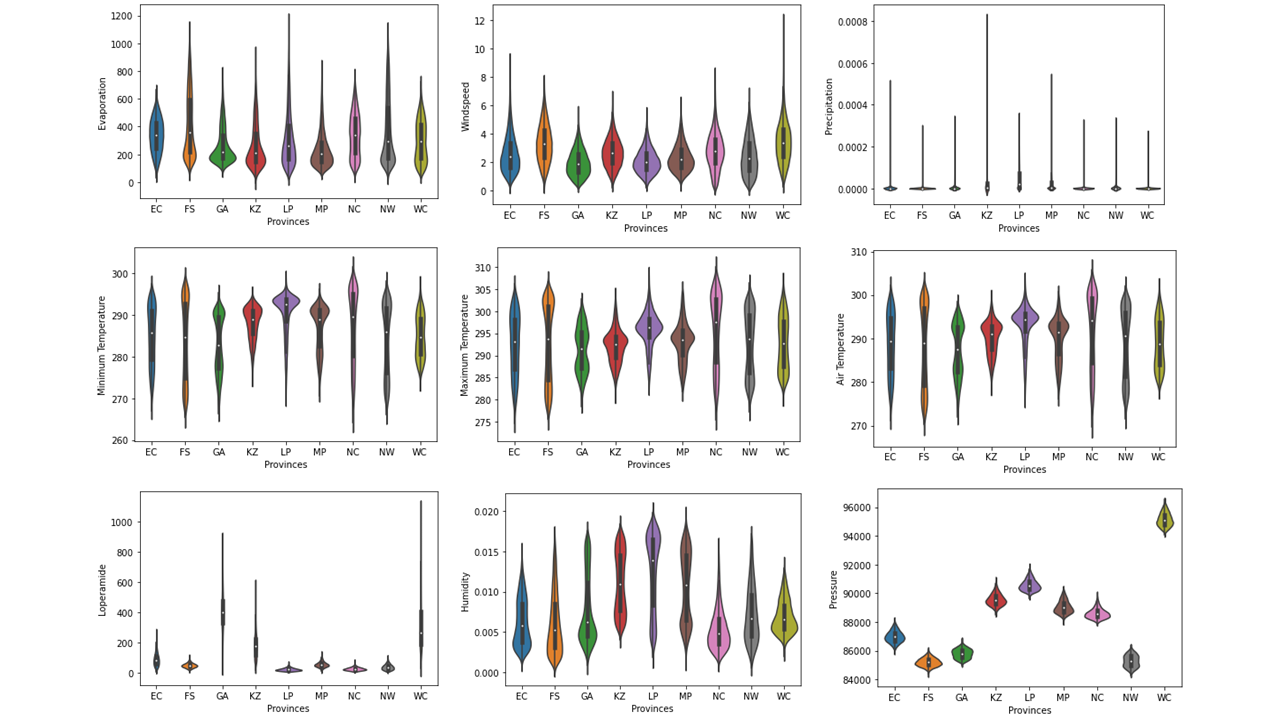

Supplement: S2 Fig — EC = Eastern Cape, FS = Free State, GA = Gauteng, KZ = KwaZulu Natal, LP = Limpopo, MP = Mpumalanga, NC = Northern Cape, NW = North West, WC = Western Cape. (TIF) [file pone.0262008.s003.tif]
